# Digital health equity: Crafting sustainable pathways

**DOI:** 10.1371/journal.pdig.0000703

**Published:** 2025-02-04

**Authors:** Robin Pierce

**Affiliations:** Faculty of Humanities, Arts, and Social Sciences, The Law School, University of Exeter, Exeter, United Kingdom; National Tsing-Hua University: National Tsing Hua University, TAIWAN

## I. Introduction

The rapid embrace of digital health technologies that hold the possibility to streamline and improve access to healthcare is generally regarded as a positive development in the provision of healthcare among increasing demand on limited resources and limited access due to mobility or infrastructure. The ability to extend the reach of the clinic through virtual visits, e-consults, telehealth, and the use of wearables for self-management and monitoring, could lead to declaring the digital turn in healthcare a likely success. The commitment to digital health equity is also growing in visibility accompanied by carefully crafted initiatives pinpointing recommended steps toward achieving this goal. The national contexts in which health inequities occur along patterns of marginalization and relative disadvantage within countries is also reflected in the global context in which low and middle income countries experience disadvantage relative to high income countries in multiple aspects of healthcare delivery [1]. When viewed together, health inequity stands to affect a significant portion of the world’s population, resulting in unnecessary loss and diminished quality of life for far too many people. As such, achieving health equity and its increasingly sizeable component of digital health equity, is of increasing concern. Fueled, in part, by social and political events, including the highly visible disparate impact of the COVID pandemic on traditionally marginalized groups, multiple sectors and stakeholders have been giving greater attention to issues of equity in health. In the context of the digitalization of healthcare, the call for timely focus on equity in health technology innovation grows as digital inequalities negatively affect those most at risk of health inequities, generally [2]. These calls have been met with some responsive action in the form of equity frameworks, guiding principles, and implementation guides, as well as rubrics for identifying factors to consider and populations most likely to be affected by digital health inequity [3–5], all of which stand to make valuable contributions to efforts to achieve digital health equity. A modification of the National Institute on Minority Health and Health Disparities (NIMHD) Research Framework specifically address the digital environment of health equity, offering a multilevel framework to address factors contributing to digital health inequity [6]. United Nations Development Programme’s Digital Health for Development Hub lists digital health initiatives in 70 countries, illustrating the multiple dimensions of achieving digital health equity and the significance of potential impacts [7]. Other agencies, including grassroots organizations and governmental agencies have articulated principles that should govern the pursuit of digital health equity, including The Global Health Government Principles emphasize process—constitution of governance boards, and engagement, and voting as essentials [8]. The United Kingdom has published a Digital Inclusion Framework, which notes that “some groups face a higher risk of being digitally excluded …; these groups also generally face a higher risk of health inequalities” [8]. The growing attention to digital health equity is encouraging; however, an essential component to achieving digital health equity that is underexplored is the aspect of sustainability, the incorporation of mechanisms that support sustained action toward achieving health equity. Some studies refer to the role of regulation as a way to embed improved design [2]; however, the multiple sources and types of inequitable impact suggest that we must expand our portfolio of possible sustaining mechanisms. The risk is that, without sustaining mechanisms, gains made from digital health equity initiatives and practices may be short term, subject to slippage if not contextually supported, or diminished by inadequately supportive processes, policies, and procedures. While recent technological and social developments have elevated the call for digital health equity, this article targets the need for greater attention to sustaining mechanisms in achieving digital health equity, and turns to regulation theory to examine possible ways to systematically consider the essential element of sustainability for achieving digital health equity.

Studies show that digital tools for health are following a pattern of much of past technological innovation in terms of delivering differential benefit [9–10], at least with regard to uptake [11]. A continuation of this pattern into the potentially transformative digitalization of healthcare arguably presents a sort of crossroads, with some seeing the digital turn in healthcare as a unique opportunity to shift a longstanding trajectory [12]. Failure to choose equity-promoting pathways now is likely to diminish opportunities for successful and timely intervention further down the road. The health mantra that “early intervention results in better outcomes” applies to the pursuit of health equity, as well, and certainly does for those experiencing poorer outcomes as a result of inequity.

## II. Dimensions of achieving digital health equity

**Fig 1:**
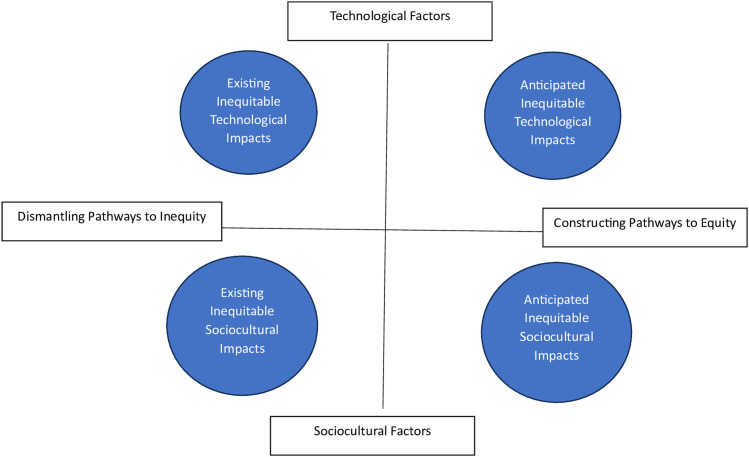
Task/source axis and categories of inequitable impacts.

The challenge of achieving digital health equity can be seen, in its simplest form, as encompassing two broad primary tasks—*dismantling* digital health inequity and *constructing* pathways to digital health equity. A recent systematic review of digital health equity frameworks and programs identified five major types of interventions being deployed—co-design, digital literacy, leveraging community/social relationships, systems-level implementation, and policies/programs targeting structural barriers [5]. Each of these types of interventions serve in either a dismantling or constructing capacity. As such, this delineation can be useful to pinpoint fundamental functions of interventions and the different challenges faced in both categories of endeavors. Rightly placed in the broader context of health equity, part of the concern about digital health inequality rests on existing and historical inequities—sociocultural aspects that are built into structures, systems, and patterns of behavior [13] that can affect how equitably a given health technology functions when used in healthcare delivery. However, digital health equity demands a greater focus on technological innovation and the extent to which it contributes to inequity, either because of its intersection with sociocultural factors or by virtue of features of the technology itself. Thus, a second spectrum refers to technological and contextual pathways to inequity. N.B. Sociocultural factors should be read broadly to encompass interpersonal, community, as well as societal and structural factors. These can be plotted as shown in (Fig 1).

The nature of tasks that need to be undertaken in order to achieve digital health equity can be viewed on a spectrum from dismantling existing pathways to digital health inequity to constructing pathways in the context of digital innovation. (Of course, some health technologies will do both in the case of displacement by a new technology that incorporates equity-promoting features regarding accessibility, ease of use, training data that is representative of target populations resulting in more equitable performance. Technological and non-technological (sociocultural) sources of inequity or technologically and non-technologically mediated sources of inequity form a second spectrum. Taking on tasks at the dismantling end of the spectrum could include such interventions as removal of consideration of race in resource allocation decisions or a refinement of metrics used to determine the allocation of life saving equipment (e.g., ventilators) [14]. Tasks situated primarily at the “constructing” end of the spectrum might include, for example, incorporating co-design for ease of use of a new technology or ensuring diversity in the training data for a new screening or diagnostic tool such that the device is more likely to function equitably.

The source of inequity also creates a spectrum that points to inequitable impacts generating from the technology itself on one end and sociocultural factors as the primary cause on the other end. (It should be acknowledged that the two are often intertwined, as in the case of technologically mediated bias. Furthermore, multiple goals can attend a single intervention.) The technological end of the spectrum could include, for example, detection technologies (e.g., light sensor) that are trained primarily on white patients, showing poorer performance in detecting the same pathology in patients of color [15]. The sociocultural end accommodates documented social determinants of health and digital determinants of health (DDOH), including low income, digital literacy, race, and infrastructure [2,6]. Of course, sources of inequity and the pathways to equity are many and complex with much overlap. This artificial division is not intended to suggest that interventions should or do take on one task or the other. To the contrary, deliberate engagement with both sets of tasks or goals, even for a single intervention, is an essential part of a necessarily comprehensive strategy toward digital health equity [2,6].

This exercise in identifying task orientation and sources of inequity is designed to facilitate analysis and discussion about possible strategies for building sustaining mechanisms into efforts to achieve digital health equity. Moreover, using these two broad dimensions of task and source sets forth four categories of inequitable impacts arising in the introduction of digital health tools, each of which presents different challenges and requires different approaches to developing sustaining mechanisms. Affording a brief consideration of common characteristics, challenges, responsibility, and possible sustaining strategies, these categories can be generally described as (1) Anticipated Technological Impacts, (2) Existing Technological Impacts, (3) Existing Sociocultural Impacts, and (4) Anticipated Sociocultural Impacts, mapping onto quadrants I–IV, respectively, as shown in (Fig 1).

*Anticipated technological impacts* (Quadrant I), high technological impact on prospective pathways to inequity, refers to a category in which the source of the inequity of concern is primarily anticipated by virtue of some aspect of the innovative technology itself (implying that some change or modification of the technology could conceivably remove biased impact). An example of an intervention taking on a Quadrant I challenge would be ensuring that a breast cancer screening tool is trained on a diverse population and can reasonably be expected to perform essential functions on historically marginalized groups, particularly those disproportionately represented in the burden of disease.

*Existing technological impacts* (Quadrant II) refers to a category in which a source of the inequity can be located in an existing technology and that the task is to disable the cause of the inequitable impact. This could involve modifying the technology, or an existing practice or procedure that embeds the technology. Features of Quadrant II impacts are that there may be evidence documenting disparate impact of the use of a particular technology, and that the use of the technology is generally accepted despite evidence of disparate impact. Dismantling the pathway to inequity could be in the form of modification of the technology. An example would be rectifying an algorithm that uses a proxy for health based on care costs, thus reflecting a longstanding racial bias in access to care in which Black patients receive less healthcare than White patients even though they may be equally ill [16]. There may be a natural inclination to observe that achieving one (dismantling) may result in creating a pathway to equity, but working with the primary or grounding task—one of displacement versus one of creation (usually pertaining to innovation) has implications for sustainability strategies, as discussed below. (Applying a counterfactual question can assist in locating the source for purposes of quadrant allocation, i.e., if the sociocultural factors were removed, would the biased impact persist and conversely, if the technology were removed or effectively modified, would the pathway of bias of concern be eliminated?) The example of the use of healthcare costs as proxy for health shows how tightly these factors can be intertwined. However, a modification in the technology could eliminate this particular resource allocation tool as a *pathway* to inequity even though it does not remove the underlying social phenomenon. As will be discussed, evidence often plays a significant role in regulation and there is generally little evidence to support claims of anticipated impact, although real-world evidence (RWE) may alter this landscape and could introduce the possibility of identifying thresholds for intervention.

*Existing Sociocultural Impacts* (Quadrant III) form a category in which the source of the inequity is primarily sociocultural or structural and the task is to dismantle or rectify some existing sociocultural aspect that contributes to inequitable impact of a digital health technology. An example of such an intervention would be a program subsidizing provision of digital infrastructure in rural communities. An example of a Quadrant III challenge would be the use of a seemingly neutral technology that, when deployed delivers disparate benefit due to sociocultural factors, and may be ameliorated by some adaptation of the “neutral” technological intervention or a change in policy or practice to minimize inequitable impact. An example of this would be a digital heart rate monitor or digital patient portals via smartphone app which may be expected to yield disparate benefit based on digital infrastructure and other DDOH.

*Anticipated sociocultural impacts* (Quadrant IV) refers to anticipated inequities upon the deployment of innovative digital health tools due to sociocultural factors. Thus, the release of a complex new smartphone application for digital monitoring of blood pressure can be expected to result in different degrees of benefit across communities due to variation in digital literacy and digital access, for example. By definition, anticipated sociocultural impacts generally offer little or no evidence of disparate impact, and has largely been the domain of translational analysis in policy or ethics or the ethical, legal, and social issues, ideally affording the opportunity to adapt the technology before deployment, as well as develop embedding protocols to minimize the impact of sociocultural inequities. These broad categories of impacts that must be addressed in order to achieve digital health equity can be used to explore strategies for developing sustaining mechanisms.

## III. Sustaining digital health equity

The implications of delineating these four categories of digital health equity challenges are particularly significant for thinking about how to attend to the need for sustained progress toward digital health equity. As forward steps are made in the development of (digital) health equity strategies, it is essential to give greater consideration to what happens to equity gains when there is no sustaining framework that supports their implementation and ongoing traction.

Exploring the challenge of achieving digital health equity must eventually engage with how to build supportive infrastructures that will facilitate sustained health equity. This usually brings the discussion to law and regulation. However, a traditional reading of regulation presents a narrow set of options for exploring sustaining infrastructures. It is helpful to turn to regulation theory, which is a distinct field that sets forth hypotheses about *why* regulation emerges, who is involved, patterns of interactions as well as *how* to regulate—which instruments and techniques are used, for which purposes, and under which conditions [17]. Of particular relevance to digital health equity is that in regulation theory the term, “regulation” is understood as a much broader concept extending beyond “legally-enforceable measures”. Instead, regulation refers to “the sustained attempt to influence behavior” [18–19], with law as but one of four primary modes of channeling behavior. Thus, the task with regard to digital health technology is to influence collective behavior in a sustained fashion. This understanding of regulation offers a wider scope for examining possible mechanisms that could contribute to building sustaining infrastructures for equity-promoting behaviors. In the following section, I briefly outline how various regulatory modalities may provide a useful platform for crafting sustainable pathways to digital health equity.

It should be noted that the notion that achieving digital health equity first is the priority and only then worry about maintaining it, would be short-sighted and vulnerable to slippage if not adequately supported and embedded in practices and policies, as well as deployed in the context of complementary equity-promoting technologies. Furthermore, gains made in one area voluntarily, e.g., a more costly equity-promoting technology design, may lose ground to more cost-competitive technologies that are designed without regard for equity impacts. Without sustaining mechanisms, such gains may be short-lived or eventually nullified by market and other forces.

The general ways that governments and other entities seek to influence behavior *in a sustained way* fall into four categories: (1) law, (2) the market (financial incentives or disincentives), (3) social norms, and (4) code or architecture [20]. This understanding of regulation is hugely significant for achieving health equity in a sustained way.

Law and regulation are perhaps most familiar, but like all four modalities, the law has disadvantages, not least the length and complexity of the legislative process. As well, the proposal to extend application of Section 1557 of the anti-discrimination law in the United States (US) to clinical algorithms met with concerns about “a chilling effect on algorithm development and use” [21]. The *market (financial incentives and disincentives)*, has recently been used in the effort to achieve health equity. In 2023, the US government agency Health and Human Services, utilized financial incentives in a scheme to make healthcare more inclusive by incentivizing the delivery of healthcare to designated underserved populations [22]. Governments have achieved significant change in collective behavior by this means, with familiar examples including cigarette and alcohol taxes. Moreover, beyond the “pinch” of higher prices, the long view is that the financial dis/incentives will inspire the desired behavior (reduced smoking and drinking) in a lasting way, contributing to the development of norms of engaging in equity-promoting behavior. Of course, not all behaviors are modifiable by financial consequences, and the elasticity of abstaining from equity-promoting behaviors is yet to be established. In general, financial incentives offer the benefit of both short-term gain (to those engaging in the incentivized behavior as well as for its possible longer-term impacts, including by way of any infrastructure that may attend equity-promoting modifications) [22].

Designing a technology or artifact such that it facilitates or limits a targeted behavior, referred to as “code” or “architecture”, has received considerable attention with regard to digital health technology, as illustrated by “privacy by design” and explainable AI. Largely obviating the need for human oversight and enforcement, “techno-fixes” as a way of influencing behavior can offer considerable efficiencies. Nevertheless, it can be a be a blunt instrument, subject to over- or under-inclusivity if not perfectly targeted toward all permutation of the undesired/desired behavior, yet it affords an immediate interruption of undesired behavior in most instances and, if done at scale, could well serve efforts toward digital health equity.

Lastly, social norms influence behavior by relying on informal social censure. This mechanism is likely to be an important component in achieving digital health equity and, like equity in many other domains, such as employment, changes in norms play a substantial role in building sustainability of equitable modes of behavior or action. With monitoring and enforcement built in, social norms deliver a relatively rapid “penalty” for non-compliance without the need for litigation. Responsible bodies in public health and beyond have successfully shifted social norms with regard to several behaviors, including smoking and littering. Although standards can transverse modalities, they may be most effectively situated in discussions of norms. Arguably, a form of social norms to influence behavior in technological development can be found in the field of standardization in community consensus of good practice [23]. Adaptable to multiple modalities, standards may be of particular interest in the context of digital health equity. Indeed, the standardization bodies in Europe and the US are in various stages of developing equity standards for AI, generally [23,24].

The many initiatives, programs, and tools being developed to further digital health equity in recent years is encouraging, and measurable movement toward digital health equity should be anticipated with optimism. However, the need to reinforce and complement ongoing strategies with supportive sustaining mechanisms should not be overlooked. Drawing on various established ways of influencing behavior in a sustained way, greater attention can be given to developing sustaining infrastructure for digital health equity.

### Developing sustaining mechanisms for digital health equity

Developing sustaining frameworks for digital health equity will depend on the nature of the task to be undertaken in the effort to achieve it. In other words, sustaining interventions that dismantle existing pathways to digital health inequity are likely to be different from those seeking to support sustained equity-promoting innovation. Proposals for evidence-based equity interventions are likely to draw the attention of policymakers in ways that proposals seeking to address anticipated inequitable impacts may find challenging. As such, a focus on categories of challenges as outlined above can be helpful in crafting sustainability strategies. With the goal of supporting sustained efforts to influence equity-promoting behavior and actions, a brief exploration of using multiple regulatory modalities to anchor sustainability strategies for digital health equity can illustrate areas for future research and development.

### Sustaining digital health equity: Addressing anticipated inequitable impacts

Much attention regarding digital health equity has been devoted to anticipated impacts based on features and design of a technology or sociocultural factors, which may lead to inequitable benefits. The anticipatory aspect of influencing behavior in the development of health technology is generally guided by assessments of safety and risk. As such, inequitable impacts likely to stem from an innovative technology have generally not been a basis for regulatory intervention [21]. If a digital health tool has an acceptable risk profile and is deemed safe, beyond voluntary engagement in equity-promoting innovation, there are currently few mechanisms that motivate and sustain equitable digital health innovation. Even if some innovators voluntarily incorporate an appropriate digital health equity framework to produce equity-promoting health technologies, absent a sustaining regulatory infrastructure, competition and other market forces could undermine those efforts over time.

When sociocultural factors such as access or digital literacy can be expected to operate to cause a digital health tool to create a pathway to inequity, in some instances the technology can be modified to minimize inequitable impacts and, along with participatory design, ways of compensating for sociocultural factors may be adopted either in the technology or its embedding. Many health equity frameworks offer specific queries to facilitate identification and address of sociocultural factors contributing to health inequity. The need for sustaining mechanisms for such equity-promoting interventions could conceivably include financial incentives as reflected in adoption and reimbursement policies of digital health tools that specifically target underserved populations, particularly where underserved populations carry a disproportionate burden of disease. This could prove particularly relevant in addressing global inequities in digital health, as tools for managing HIV or other infectious diseases and increased access to infrastructure and remote health service delivery through telehealth, drones, and other smart devices [1] become increasingly effective. In addition to market mechanisms, changing norms will need to play a role in addressing anticipated sociocultural impacts such that consideration and adaptation minimizing inequitable sociocultural impacts is the default. Where technological design can address anticipated inequitable impacts effectively, standards may offer one way of shifting norms, and, if the underlying consensus is sufficiently robust, perhaps even triggering a norm cascade [25], in which adoption and utilization of a norm, in this case an equity standard, quickly becomes the expected way to develop a digital health tool (and tools without the standard are considered less desirable). The crafting of the incentives such that they accurately and precisely target the desired or undesired behavior is critical. Similarly, a well-crafted incentive can achieve low maintenance changes in behavior at scale. This may be a particularly appealing regulatory option for incorporating equity into design, particularly if accompanied by clear metrics and measurability according to a relevant evaluation plan.

### Sustaining digital health equity: Addressing existing inequitable impacts

The challenges brought by existing inequitable technological and sociocultural impacts present a different profile of sustainability needs. The act of displacement or dismantling an existing practice, technology, or procedure is, at a minimum, a matter of evidence, better alternatives, and political will. Altering existing technology or its embedding, in the case of sociocultural factors, will necessarily affect persons or institutions with an interest in the status quo. The options for legal measures may be greater depending on the strength of the evidence and the nature of a contemplated intervention. If there are technology alternatives that produce more equitable outcomes, market measures pertaining to adoption and reimbursement, may be appropriate. As mentioned, compliance with norms in the form of standards that could be achieved through modification or replacement of existing technology could also serve to sustain digital health interventions seeking to dismantle pathways to inequity.

In the case of dismantling existing sociocultural factors, creativity and adaptation may increasingly play a role in altering pathways to inequity. Equity-promoting technological modification responding to existing sociocultural inequities can be incentivized (as described above for anticipated sociocultural impacts). Sociocultural factors can be re-examined in light of participatory research on existing and prospective digital health use, serving to better identify sociocultural barriers based on local knowledge. Mechanisms that could help sustain such practices will need to ground in norm-shifting strategies, for example, regarding requirements in grant applications or checklists for research ethics forms. Sustaining frameworks for digital health equity efforts addressing inequitable technological and sociocultural factors should be oriented toward shifting the norm—the expectation that innovation should deliver equitable impacts, and where deviation from that needs to be justified. The recently issued FDA guidance on diversity in recruitment to clinical trials, which requests specific information pertaining to diversity and requires justification for non-compliance, is an example of how such a sustaining mechanism can be crafted [26]. Nevertheless, this kind of non-compulsory guidance requires, at the very least, an embedding that elevates the importance of compliance even when it is not legally mandated.

## IV. Conclusion

Traction for equity gains must involve a change not only how we regard health equity in innovation, but what we think should be done about it and by whom. The need to develop sustaining mechanisms for digital health equity regarding anticipated inequitable impacts should not be underestimated. Although evidence of disparate impact is generally lacking in the case of anticipated inequitable impacts, the role of RWE may allow for new approaches to preventing inequitable impacts earlier in the deployment cycle. Additionally, closer examination is needed of the role of evidence in supporting the introduction of equity-sustaining mechanisms utilizing the four modalities mentioned above. Crowe and Rodriquez recently published an article in JAMA in which they concluded that achieving digital health equity is the “responsibility of all of us” [27]. This observation has multiple dimensions and underscores the need for developing sustaining mechanisms that also work to shift norms to more robustly center equity in the introduction of digital health tools. As reliance on new digital health tools increases, continued or escalation of disparate benefit operating to the relative disadvantage of the already marginalized could have significant impacts on the health and quality of life of much of the world’s population. How to move from a relentless history of health inequity to an evolving realization of the promise of digital health equity requires that we see this challenge as an opportunity.
